# Enhancing Targeted Therapy in Breast Cancer by Ultrasound-Responsive Nanocarriers

**DOI:** 10.3390/ijms24065474

**Published:** 2023-03-13

**Authors:** Isaiah A. Edwards, Flavia De Carlo, Juliana Sitta, William Varner, Candace M. Howard, Pier Paolo Claudio

**Affiliations:** 1 Department of Radiology, University of Mississippi Medical Center, Jackson, MS 39216, USA; 2 Department of Pharmacology and Toxicology, Cancer Center and Research Institute, University of Mississippi Medical Center, Jackson, MS 39216, USA

**Keywords:** ultrasound, enhanced targeted therapy, ultrasound-sensitive carriers, breast cancer

## Abstract

Currently, the response to cancer treatments is highly variable, and severe side effects and toxicity are experienced by patients receiving high doses of chemotherapy, such as those diagnosed with triple-negative breast cancer. The main goal of researchers and clinicians is to develop new effective treatments that will be able to specifically target and kill tumor cells by employing the minimum doses of drugs exerting a therapeutic effect. Despite the development of new formulations that overall can increase the drugs’ pharmacokinetics, and that are specifically designed to bind overexpressed molecules on cancer cells and achieve active targeting of the tumor, the desired clinical outcome has not been reached yet. In this review, we will discuss the current classification and standard of care for breast cancer, the application of nanomedicine, and ultrasound-responsive biocompatible carriers (micro/nanobubbles, liposomes, micelles, polymeric nanoparticles, and nanodroplets/nanoemulsions) employed in preclinical studies to target and enhance the delivery of drugs and genes to breast cancer.

## 1. Introduction

Breast cancer is the most common cancer in women in the United States and accounts for 30% of all newly diagnosed invasive cancers in the female population. The American Cancer Society’s 2022 estimate put the number of cases of newly diagnosed invasive breast cancer at 287,850. About 43,250 women will die from breast cancer making it the second leading cause of cancer death in women, only after lung cancer. Despite the significant decline in the death rate between 1989 and 2019, death from breast cancer is 41% higher in Black women than in White women, in part due to the higher proportion of triple-negative breast cancer diagnosed and less access to high-quality cancer care [1]. The majority of breast cancer cases might be attributed to factors linked to pregnancy, hormonal therapy, and lifestyle [2]. Approximately 10% of all cases are related to genetic predisposition, family history, and ethnicity. Germline mutations in BRCA1 and BRCA2 genes are most commonly associated with breast cancer, with an average cumulative lifetime risk of about 70% [3,4]. Historically breast cancer has been classified into three subtypes based on the expression of estrogen (ER) or progesterone (PR) hormone receptors and human epidermal growth factor receptor 2 (HER2/ERBB2). Specific therapeutics can be used to target these subtypes. Triple-negative breast cancer (TNBC) does not express any of these markers and is treated with conventional chemotherapy and radiation therapy [5,6,7,8]. Twenty years ago, Perou and Sorlie performed expression-profiling studies that showed how ER/PR/HER2 classification is not sufficient to depict the heterogeneity of breast cancer. Five main breast cancer subtypes have been identified: basal-like, HER2-enriched, luminal A, luminal B, and normal-like [9,10,11]. Luminal A tumors are ER/PR-positive and HER2-negative, slow growing and often of lower grade, and have a good prognosis. They are treatable with hormonal therapies [12,13]. Luminal B tumors have a slightly worst clinical outcome than luminal A tumors. They are usually ER-positive and may be either HER2-positive or express high levels of Ki67, which makes them fast growers. The latter subtype is treatable by hormonal therapy but frequently relapses [14,15,16]. HER2-enriched breast cancers are driven by ERBB2/HER2 gene amplification, and they grow faster than luminal cancers. They have a worse prognosis, but they can be targeted with anti-HER2 monoclonal antibodies and HER1/EGFR/ERBB1 and HER2 kinase inhibitors [16,17]. Basal-like tumors are ER-negative and triple-negative breast cancers (ER-, PR-, and HER2-negative) that can considerably overlap with basal-like tumors even if they are distinct from a clinical and histopathological standpoint [18,19]. These tumors are not treatable with hormonal and/or aromatase inhibitor therapies [20] and unfortunately, chemotherapy achieves limited clinical response, high toxicity is often observed, and patients can develop multidrug resistance. The high molecular heterogeneity, risk of metastasis and relapse, and association with BRCA1 mutation are additional factors that contribute to poor prognosis and management of TNBC [21,22,23,24,25,26]. 

## 2. Breast Cancer Treatments and Treatments’ Limitations

Breast conservation treatment and mastectomy are well-established local therapies for invasive breast cancer [27,28,29,30,31]. Regardless of the type of surgery performed, local recurrence rates differ considerably among subtypes, and they are highest among TNBC [32,33]. The delivery of adjuvant whole-breast irradiation following lumpectomy decreases recurrence rates by about 50% and increases survival [27,28,29,30]. Post-mastectomy radiation instead is a well-established component of breast cancer treatment in patients with advanced disease with more than four positive lymph nodes and tumors larger than 5 cm [34]. Following surgical resection of the tumor, patients often receive adjuvant systemic therapy to eradicate undetected micrometastasis that is selected depending on the disease burden (the number of lymph nodes and the size of the primary tumor) and ER/PR/HER2 status, and genomic assays. Neoadjuvant chemotherapy was originally used to make inoperable breast cancer resectable, but it has also been used with the intention of facilitating breast conservation. A standard chemotherapy regimen usually contains anthracycline (doxorubicin) and taxane (paclitaxel and docetaxel) [35,36,37,38]. Hormonal therapy is recommended for most ER/PR-positive breast cancer patients with tamoxifen being used in both pre- and postmenopausal and aromatase inhibitors (anastrozole, letrozole, and exemestane) only used in postmenopausal women [39]. Patients may be treated with endocrine therapy for 5–10 years or more with additional benefits observed with longer therapies [40,41,42]. Patients with HER2-positive breast cancer are given HER2-targeted therapy in combination with chemotherapy. Patients with stage I cancer often receive paclitaxel and anti-HER2 monoclonal antibody trastuzumab [43] and those at stage II-III receive two HER2-targeting agents, trastuzumab and pertuzumab, (HER2 dimerization inhibitor) [44,45]. The lack of ER, PR, and HER2 receptors in triple-negative breast cancer eliminates the benefits of endocrine therapy; therefore, chemotherapy is the treatment of choice [20]. 

## 3. Conventional Chemotherapy, Nanomedicine, and Enhanced Permeability and Retention Effect (EPR)

By standard systemic delivery, chemotherapy reaches untargeted organs often leading to undesirable side effects [46]. Drug toxicity, tumor heterogeneity, and anatomical barriers are major limiting factors to effective cancer treatment and may increase morbidity, impair quality of life, and, when severe, may be lethal [47]. The severe side effects correlated to high doses of chemotherapy and the absence of targeted strategies for drug administration, especially in the case of patients diagnosed with TNBC, demand the development of new therapeutic tools such as nanotechnology-based drug delivery [48,49,50] which can improve the biodistribution and the accumulation of chemotherapy [51]. 

**Figure 1:**
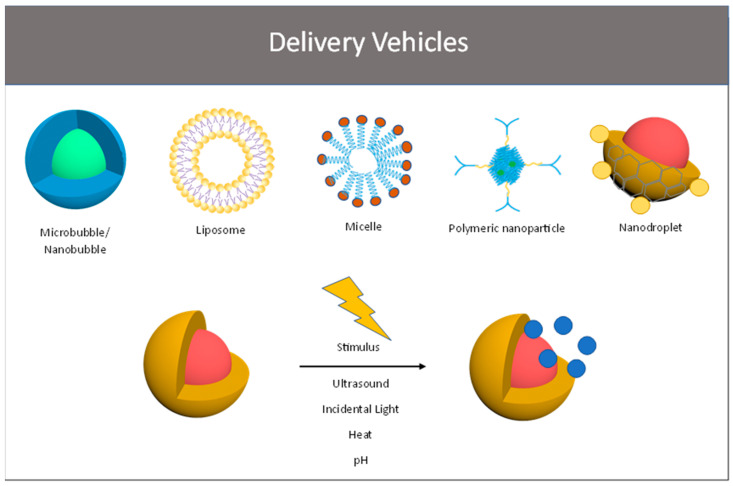
Graphical abstract of delivery platforms.

Various delivery systems have been designed recently with the aim of reducing the general toxicity of conventional chemotherapeutics and increasing the therapeutic index of these drugs. Figure 1 shows the delivery platforms that will be discussed in this review.

Conventional chemotherapeutic drugs, such as doxorubicin and cisplatin, have a short blood half-life, conspicuous off-target accumulation, and an unspecific mechanism of action [52]. A notable increase in the plasma half-life of doxorubicin was obtained by encapsulating the drug in liposomes (Caelyx^®^/Doxil^®^) [53] and by modifying the surface with polyethylene glycol (PEG) to decrease aggregation and opsonization reducing mononuclear phagocyte system uptake (stealth liposomes) [54,55]. Extended systemic circulation allows for these nanomedicines to accumulate in the tumor site by passive targeting due to its pathophysiological characteristics. The enhanced permeability and retention (EPR) effect observed in tumors versus healthy tissues and first described by Matsumura and Maeda in 1986 [56] is a result of leaky vasculature and poor vessel perfusion [57,58,59,60]. The extent of the EPR effect depends not only on the growth of a chaotic vasculature but also on the tumor microenvironment [59]. The low or absent functional lymphatic vessels that result in high interstitial fluid pressure, the presence of deregulated stromal cells, and abnormal and overexpressed extracellular matrix made of a cross-linked network of hyaluronic acid, elastin fibers, collagen, and proteoglycans can reduce the diffusion of the nanodrugs in the interstitium. All these factors affect the even distribution and accumulation of nanoparticles in the tumor, leading to a clinical outcome that is highly heterogeneous [61,62,63]. Despite the possibility of functionalizing the nanoparticles to target over-expressed receptors (e.g., VEGF, EGFR, and HER2) [64,65], it has been reported in a meta-analysis by Wilhelm et al. that only 0.7% of the administered therapeutics can be targeted to the solid tumor solely by the EPR effect [66,67].

## 4. Ultrasound

One approach that has been developed to increase the EPR effect is the use of external stimuli such as ultrasound which can enhance the permeability of the blood vessel and tissues and increase the release of drugs from the carrier.

Mammography, ultrasound (US), magnetic resonance imaging (MRI), and positron emission tomography–computed tomography (PET/CT) are the main imaging tools available for breast cancer screening, diagnosis, staging, surgical planning, and surveillance [68]. Additionally, diagnostic ultrasound is used as procedure guidance in a wide variety of clinical settings [69].

Currently, three ultrasound contrast agent compositions have been approved by the Food and Drug Administration (FDA) and the European Medicine Agency (EMA) for diagnostic purposes: SonoVue/Lumason, Definity, and Optison. SonoVue (Bracco Imaging S.p.A., Colleretto Giacosa, Italy) and Lumason (Bracco Diagnostics Inc., Monroe Township, NJ, USA) are sulfur hexafluoride lipid-type A microspheres constituted of polyethylene glycol 4000 and phospholipids DSPC (1,2-Distearoyl-sn-glycerol-3-phosphocholine) and DPPG (1,2-Dipalmitoyl-sn-glycerol-3-phosphoglycerol, sodium salt) [70]. The EMA approved the use of SonoVue to improve the display of the vascularity of liver and breast lesions during doppler sonography in adult patients leading to more specific lesion characterization [71]. Definity (Lantheus, North Billerica, MA, USA) are perflutren (Octafluoropropane) gas lipid microspheres constituted by three phospholipids: DPPC (1,2-Dipalmitoyl-sn-glycerol-3-phosphocholine), DPPA (1,2-Dipalmitoyl-sn-glycerol-3-phosphate, sodium salt) and DPPE-MPEG5000. Optison (GE Healthcare, Oslo, Norway), perflutren (octafluoropropane) gas protein-type A microspheres (USP, GE Healthcare, AS, Oslo, Norway) are constituted by a human serum albumin shell [70].

The main advantages of using US include wide availability, low cost, no ionizing radiation, and real-time dynamic imaging capability. Lower spatial resolution and tissue penetration are instead disadvantages [72]. Besides its diagnostic utility, ultrasound in combination with contrast agents or ultrasound-responsive nanomedicines has been more recently explored as a tool that can enable the direct visualization of a tumor, and guide and enhance the delivery of therapeutics to the targeted region using thermal and mechanical effects.

## 5. Thermal and Mechanical Effects of US

Acoustic waves interact with body tissues, cell membranes, and drug carriers via a combination of thermal and mechanical effects. US generates compression and rarefaction pressure alternations at different frequencies. Typically, higher frequencies (>20 MHz) utilized for diagnostic purposes have lower tissue penetration (i.e., higher attenuation), while lower frequencies (0.5–5 MHz) used for therapeutic applications enable deeper tissue penetration [73]. Adjustments in the ultrasound settings (the mechanical index, pulse repetition frequency, etc.) allow for several biologic effects such as thermogenesis, cavitation, and acoustic radiation force [74]. Thermal effects are explained by the conservation of energy law, which is in part absorption of sound waves by imaged tissues. Utilizing this phenomenon allows for noninvasive, fast, and localized heating of deeply located tissues [74]. The degree of tissue thermogenesis is set by US parameters such as the thermal index, transducer geometry, and sonication frequency. Greater degrees of thermogenesis may be reached with a high-intensity focused ultrasound (HIFU) that can achieve a tea temperature >60 °C and that is used in clinical practice for several ablative treatments such as uterine fibroids, bone metastases, and prostate cancer [75]. The level of hyperthermia obtained with ultrasound (40–45 °C) has been shown instead to enhance cell membrane fluidity, increase permeability to drugs, and also lead to the release of drugs from thermosensitive carriers without tissue damage [76,77,78]. Direct disruption of the target membrane occurs not only directly with membrane fluidification, but also by sonoporation, in which microbubble implosion disrupts neighboring cell membranes temporarily [79,80]. This allows direct communication between the cytoplasm with external tissue environments, allowing for macromolecules or nanoparticles to be directly delivered. This principle was utilized by Dewitte et al. in the delivery of immunomodulatory TriMix mRNA in addition to a desired antigen mRNA incorporated into microbubbles to educate and mobilize antigen-specific T cells in destroying the transformed target cells. Additionally, Domenici et al. leveraged low-intensity ultrasound-induced sonoporation to deliver gold nanocolloids conjugated to 4- aminothiphenol, an infrared marker, into murine fibroblasts, with significant cytotoxic and genotoxic effects after ultrasound/nanocolloid combination [81].

Acoustic cavitation describes the process of alternate compression and rarefaction of a solid or liquid in a medium that conveys acoustic irradiations, a phenomenon described and utilized with US contrast agents, the microbubbles (MBs) [82]. This oscillation of volumetric expansion and contraction has numerous effects: volumetric expansion of a spheroid greatly reduces the pressure of the phase contained therein, generating negative pressure. The subsequent collapse of the spheroid reduces volume, which increases the pressures and temperatures, the latter of which is imparted into the surrounding fluid phase [83]. Cavitation may be classified as non-inertial (also known as stable) or inertial (also known as transient). Non-inertial cavitation describes the stable compression and expansion oscillation of MBs, without collapse. This process generates a secondary effect known as microstreaming, the local turbulence of particles near the endothelial lining that promotes endothelial permeability and, thus, increases local drug delivery [84]. Inertial cavitation, on the other hand, involves exaggerated MBs oscillation, with a marked unbalanced expansion phase and ultimately MBs collapse. Additionally, inertial cavitation leads to microstreaming, microjet, free radical formation, shear wave, and local thermogenesis [82]. Acoustic radiation force (ARF) consists of primary and secondary effects. The primary effect moves particles away from the transducer and the secondary effect promotes attraction between particles [85]. ARF pushes therapy or its carriers away from the center of the vessel towards the endothelial wall. Summed to the additional above-mentioned US effects, this phenomenon is an important adjuvant in US-mediated targeted drug delivery [86,87].

The cellular effects of ultrasound exposure have been well documented. Most damage to irradiated tissues is caused by exposure above the cavitation threshold, where oscillating pressures induce the formation of micron or smaller gas bubbles, which with the resulting oscillation and collapse, induce severe damage in the irradiated cells [88]. However, the damage is not limited to energy levels exceeding this threshold, and this is important to consider when balancing potential damage from higher levels of US irradiation with enhancement in membrane permeability/integrity for maximizing the delivery of desired materials. Recent work highlights the biological effects of sub-cavitation threshold irradiation in keratinocytes. Increasing doses of ultrasound exposure produced decreases in cell viability and the activation of apoptosis [81]. Moreover, at higher doses, overexpression of IL-6 was observed, which was thought to occur through ultrasound imparting mechanical stress within the cell [89]. These findings illustrate that even below the commonly accepted threshold where most damage occurs, ultrasound can impart damage to tissue that can reduce viability, increase inflammatory changes, and potentially have untoward off-target effects on target tissues.

Additionally, ultrasound can directly interact with the carriers leading to the release of therapeutic drugs in the region targeted by the ultrasound. It has been shown that HIFU can open ultrasound-sensitive lock copolymer polyethylene glycol (PEG) and polypropylene glycol (PPG) micelles, leading to the destruction and release of the payload [90]. The degradation behavior of hollow PLGA poly(lactic-co-glycolic acid) (PLGA) contrast agents microcapsules was studied by El-Sherif et al. They showed that microcapsules that are more echogenic degrade faster than those that are less echogenic and this is further accelerated by using an ultrasound frequency that gives maximum backscatter [91].

The ability of ultrasound to induce the delivery of therapeutics to a target with the myriad of vehicles under development is a highly complex interplay between target tissue/tumor porosity, either induced or native, penetration of ultrasound to the target of interest, and the interaction of the physiochemical properties of the vehicle with the exposure parameters of ultrasound. These parameters can be highly variable depending on the complex interplay of biological and mechanical effects discussed above. For instance, micelles have been explored at a variety of exposure parameters with interesting results. Husseini et al. illustrated that pluronic micelles loaded with doxorubicin had their most efficient drug release at 20 kHz which decreased reliably with increasing frequencies despite the associated increase in power density, which suggests that inertial cavitation and its subsequent mechanical effects on local tissues is a large driver of drug delivery in some micellar systems [92]. Furthermore, dual frequency acoustic radiation with similar doxorubicin loaded pluronic micelles at 27.7 kHz at 0.02 and 0.04 W/cm^2^ and 3 MHz at 1 and 2 W/cm^2^ revealed significantly higher doxorubicin offloading than either exposure parameter alone, which suggests that the local thermal effect of higher intensity ultrasound likely also dictates drug delivery in part [93]. Liposomes have also been shown to efficiently offload their payloads at low-frequency ultrasound irradiation, at frequencies as low as 20 kHz, which is believed to be a function of transient porosity in the liposome’s bilayer [94]. Concerning microbubbles, jet formation has been illustrated to form at frequencies as low as 1 MHz, suggesting that inertial cavitation can occur at relatively low energy densities [95] and rigid-shelled microbubbles have been reported to undergo at least partial cracking at frequencies of 0.5 MHz, although an increased proportion of cracked rigid-shelled microbubbles occurred by increasing the frequency to 1.7 MHz [96]. Mannaris et al. highlighted further implications of varying acoustic radiation exposure when they characterized the extravasation trends and penetration depth of gas-trapping nanoparticles, microbubbles, and nanodroplets [97]. They illustrated that higher frequencies of ultrasound (1.6 and 3.3 MHz vs. 0.5 MHz) generated strong directional extravasation away from the ultrasound source, and increasing exposure time as well as discrete ultrasound pulse length produced increased amounts of extravasation of gas-trapping nanoparticles, microbubbles, and droplets. Of these, gas-trapping nanoparticles were found to have the highest amount of extravasation for the lowest energy density when compared to microbubbles and droplets. Ultimately, the exact mechanisms that interplay to create optimum vehicle extravasation and eventual therapeutic delivery to target tissues are still being elucidated, and optimum exposure parameters are likely highly specific to the physiochemical properties of a vehicle and its design philosophy. Continued work to characterize both delivery vehicles’ performances at various exposure parameters, as well as the underlying phenomena that coalesce to dictate effective therapeutic delivery, is necessary to further develop effective therapies.

## 6. Ultrasound-Sensitive Micro- and Nanocarriers

In this section, we will discuss some of the ultrasound-sensitive micro and nanocarriers that have currently been employed for breast cancer preclinical research. The studies are listed in Table 1.

### 6.1. Micro- and Nanobubbles

Microbubbles (MBs) and nanobubbles (NBs) are gaseous core encapsulated US contrast agents widely used for diagnostic applications. Under diagnostic US settings, MBs contract and expand, an effect known as stable cavitation, producing prominent backscattering, which is readily identified in imaging [118]. This effect has been successfully used to assess contrast-enhanced patterns of tissues of interest in several medical applications, similar to other clinical imaging modalities such as CT and MRI. Given the above-mentioned inertial and non-inertial effects of ultrasound, extensive research has explored the effect of MBs in therapeutic applications, turning them into a potential minimally invasive theragnostic tool. In this system, US provides diagnostic imaging with real-time visualization of the targeted tissue and controlled sonication-induced therapeutic drug release [82].

The MB-NB’s shell consists of an inner layer, in contact with the core gas, and an outer layer, in contact with the outer space. The shell may be composed of a variety of biocompatible materials and combinations, the most common including protein, lipid, and polymer. The shell’s physicochemical properties are a major dictator of the MB-NBs behavior under ultrasound, shell life, stability, immunologic reaction, and drug-carrying capabilities [119,120]. NBs’ size, which is variable depending on composition but generally accepted to be sub-micron, makes them less echogenic than MBs, which are classically delineated as anything above one micron with a range of 1–10 microns in practice; however, the shell composition can be modified to improve their response to acoustic waves. More importantly, the smaller size of NBs allows for their passive accumulation in the tumor by EPR while MBs, which are a few microns, cannot cross the gaps between endothelial cells. Additionally, NBs are more stable and display a longer circulation time than MBs [121].

Protein shell bubbles are commonly made of albumin [120]. Albumin-coated encapsulated microbubbles are produced by heating the protein solution to its incipient temperature, followed by sonication. Sonication of the pre-heated protein solution further increases its temperature leading to denaturation. Denaturized protein molecules typically present cysteine residues with disulfide bonds that are broken during the denaturation process. Reaggregation of denaturized protein fragments enveloping a gas core occurs during sonication and can be achieved by modifications in the solution’s pH. For instance, at the isoelectric point, repulsive forces are eliminated, facilitating free molecule aggregation. The thick protein shell layer can accommodate the loading of macromolecules without significantly disrupting the sonication response [122]. The easiest method to incorporate genes and drugs into the protein MB is by simple incubation of the desired macromolecule with an MB solution [120,123].

Lipid-shelled MBs utilize the physiochemical properties of lipids to stabilize the MB. The hydrophilic component allows contact with the outer space solution while the inner hydrophobic layer keeps the gas core entrapped. Efficient gas entrapment also demands a cohesive bound and a high-density shell layer. The lipid layer is bound by hydrophobic interactions. To achieve a compact lipid layer, the solution undergoes heating followed by rapid quenching [124]. Since lipid molecules are bound by weak hydrophobic and Van der Waals interactions, lipid MBs easily undergo expansion and resemblance under US cavitation. The thin lipid layer, however, limits the loading capacity. Different strategies have reported incorporating macromolecules into the outer layer as well as into the hydrophobic layer without altering MBs’ response to ultrasound [120,125]. Different from protein-shelled MBs, lipid shells rely on ultrasound inertial cavitation with induced fragmentation, microjet formation, and microstreaming [82].

Synthetic and natural polymer-based MB shells claim better control of the composition and elasticity of the shell, potentially providing a more stable and predictable behavior under ultrasound. There are several methods described to prepare polymer-encapsulated MBs, including internal loading with the gas core, physical association, and covalent linkage with the polymer shell [126,127,128]. Polymer-shelled MBs may also be developed to increase circulation time and provide a higher ligand density [129]. Polymer-shelled MBs present physicochemical properties that are different from the lipid monolayer. The compact polymer layer is less compressible, may withstand a non-spherical shape, and undergoes sonic cracking under US, a process where the MB capsule cracks resulting in anisotropic gas core release [96,130,131].

To illustrate the feasibility of polymeric MB shells, Oddo et al. [132] designed a system using poly (vinyl alcohol) (PVA) microbubbles and incorporated robust multifunctionality by conjugating superparamagnetic iron oxide nanoparticles, a near IR reporter, a cyclic arginyl-glycyl-aspartic acid (RGD) peptide, and cyclodextrin to improve vector targeting and drug delivery. PVA-RGD surface conjugation allowed for the selective recognition of αVb3 integrins preferentially expressed in the neovascularized endothelium. The RGD-conjugated microbubbles were illustrated to have vigorously increased adhesion compared to a control. Additionally, cyclodextrin incorporation into the bubble shell permits the potential to load hydrophobic drugs into the MB’s core in the absence of covalent bonding, which allows for the delivery of normally poorly soluble drugs to the target tissue. With this system, they obtained 24 h of controlled release of dexamethasone acetate, normally poorly soluble. Very recent work by Da Ross et al. illustrates a growing scope for surface functionalized PVA microbubbles with a system such as the previously described RGD/integrin binding system, increasing their scope to radioembolization with a yttrium payload to treat glioblastoma multiforme [133].

Recently, microbubbles have even been used to explore circumventing tumor hypoxia, a poor prognostic factor in determining radiotherapy sensitivity, by being loaded with an oxygen payload in attempt to oxygenate the tumor microenvironment for better response to radiation therapy. Fix et al. constructed 1,2-distearoyl-sn-gylcero-3-phosphocholine (DSPC) microbubbles loaded with oxygen to target rat fibrosarcoma in vivo [134]. The results illustrated higher oxygen levels at the site of the tumor and increased responsiveness to subsequent chemotherapy, which is an exciting prospect for the scope of microbubbles as an adjuvant in hypoxic cancers. Further optimizing this system, Reusser et al. loaded 1,2-dibehenoyl-sn-glycero-3-phosphocholine (DBPC) and DSPC microbubbles with oxygen and assessed for contrast enhancement and kinetics [135]. The longer acyl chained microbubbles, DBPC, showed superior contrast enhancement and circulation times in vivo, representing an optimization on previous renditions of phospholipid oxygen microbubbles.

#### 6.1.1. MBs/NBs for Breast Cancer Treatment

Morch et al. developed a new multifunctional delivery system consisting of microbubbles for ultrasound stabilized by PEGylated nanoparticles of poly (butyl cyanoacrylate) PBCA polymer. By applying an appropriate ultrasound pulse the bubble can burst and the NPs containing drugs can be released into the tumor region [136]. By using this platform, Snipstad et al. tested the effects of US-enhanced cabazitaxel release in breast cancer and they showed a 2.3 tumor uptake improvement after bubbles destruction by increasing focused mechanical index that directly correlated with increased intra-tumor nanoparticle deposition [98].

Using a different approach, a dual-modal microbubble containing SF6 gas and consisting of lipid microbubbles loaded with paclitaxel and functionalized with RGD (tripeptide Arg-Gly-Asp) to specifically target tumors was developed. The application of ultrasound allows for an increase in drug accumulation in TNBC-targeted tumors in vitro. These microbubbles are constructed with the following biocompatible materials: RGD-PEG-DPPE (1,[2-dipalmitoyl-sn-glycerol-3-phosphoethanolamine]-N-[amino (polyethylene-glycol)]), DPPC (1,2-dipalmitoyl-sn-glycerol-3-phosphoethanolamine), and cholesterol [99].

The use of ultrasound-mediated nanobubble destruction (UMND) was explored by Jing et al. to enhance the targeted delivery of EGFR-targeted siRNA (siEGFR) in TNBC. They synthesized NBs (DPCC, DSPE-PEG2000-MAL, DPPA, and PEG-40 stearate) loaded with cell-penetrating peptide (CPP) and carrying siEGFR and utilized US to deliver siEGFR into TNBC cells observing a reduction in the expression of EGFR at mRNA and protein levels together with a reduction in cell proliferation in vitro and inhibition of tumor xenografts growth in vivo [137].

It has been shown by gene expression profiles of TNBC that LINC00511 expression is significantly increased and plays a major role in cancer biology such as conferring drug resistance. UMND was used by Yuan et al. to enhance the transfer efficiency to TNBC cells in vitro of CPP-loaded nanobubbles (perfluoro-propane-filled nanobubbles synthesized using DSPC and DSPE-PEG2000) complexed with the small interfering RNA for a long intergenic non-protein coding RNA 00511- (LINC00511-siRNA). By using CPP-NBs-LINC00511-siRNA together with UMND plus, the authors showed a reduction in LINC00511 expression and increased sensitivity to cisplatin treatment [100].

Acoustic cluster therapy was employed by Bush et al. to show how microbubble/microdroplet clusters (PS101) can be used to further increase the therapeutic efficacy of Doxil in orthotopic human TNBC xenografts (MDA-MB-231-H.luc). Microbubbles/microdroplets, when exposed to low-frequency ultrasound (300 kHz) at a low mechanical index (MI = 0.15), are subjected to a phase-shift and form microbubbles of 22 µm median diameter that can transiently lodge at the microvascular level. Additional US exposure leads to bubble oscillation and increases in endothelium permeability and drug accumulation in the tumor [101].

Over 15 years ago our laboratory started to explore the use of clinically approved US contrast agents to encapsulate adenoviral vectors for cancer gene therapy. Lyophilized microbubbles were first reconstituted with a solution of adenoviruses and then treated with human complement to inactivate viruses on the bubble surface obtaining an immune stealth system. Initially, SonoVue, Sonazoid, Levovist, and Imagent MBs were tested and we showed the superiority of adenovirus encapsulation by Imagent microbubbles. This system allows for the targeted delivery of the adenovirus in the tumor region after exposure to ultrasound, expression of the transgene, and therapeutic response in vivo [138,139,140,141]. Using a similar methodology but for BC screening purposes, Warrem et al. explored the use of MBs functionalized to bind αVβ3 integrins, P-selectin, and vascular endothelial growth factor receptor-2 on the tumor vasculature, and to then release a dual-reporter adenovirus in the targeted region [142]. A different approach to accelerate the clearance and prevent the liver toxicity of adenoviruses was used by Carlisle et al. by complexing the virus with nanoparticles. This system will be discussed in the polymeric nanoparticles section [112].

#### 6.1.2. Strength, Weaknesses, and Open Issues with MB/NB

Microbubbles represent one of the first steppingstones to ultrasound-enhanced therapy, and great progress has been made in their development and optimization. The versatility of their applications and their sheer modularity, as illustrated above, are significant boons to their application in targeted therapeutics; however, limitations of microbubbles for imaging and drug delivery lie with the inability of the drug to diffuse into target tissues because their size is larger than the gaps between the vascular endothelium, and because of this, their clearance rate is accelerated [143]. While mechanisms made to increase circulation times utilizing polymeric-shelled microbubbles have been developed, future efforts to enhance the distribution or therapeutic effect will lie in minimizing size and maximizing circulation times by exploring new shell and payload configurations.

To contrast these weaknesses, nanobubbles excel where microbubbles do not. While they are not as echogenic owing to their size, their sub-micron dimensions can utilize the junctions between the endothelium to escape the vasculature and accumulate in target tissues much more readily than most MBs. Additionally, NBs are more stable and display a longer circulation time than MBs [121].

### 6.2. Liposomes

Liposomes are lipid spheres of variable size, generally ranging from 50–500 nm, composed of single or double amphipathic layers, capable of carrying internal molecules [144,145]. Like bubbles, liposomes may have their targeting capability enhanced by tagging ligands of interest to their outer surface. The lipid bilayer is composed mainly of the natural phospholipid phosphatidylcholine, a molecule with a polar hydrophilic head and two hydrophobic tails. In aqueous solutions, the phospholipid bilayer is typically oriented with the hydrophilic polar head in contact with the outer and inner spaces [146,147]. This configuration allows for the addition of either hydrophobic or hydrophilic drugs by adding molecules to the membrane surface and vesicle core or embedding them within the capsule layers accordingly. In addition to small molecules, liposomes can be functionalized with cationic moieties or lipid conjugates to deliver many combinations of genetic material, allowing for alteration, silencing, or introduction of genetic codes of target tissues to target malignancy and metastasis [148]. Cholesterol is a secondary but essential component in the liposome capsule’s stability and membrane permeability modulation [149,150]. Further capsule modifications to improve the biodistribution profile may include the addition of PEGylation, membrane proteins, such as site-specific antibodies, and polymers [151,152]. Since these biocompatible drug carriers have a similar composition to membrane cells, they are capable of fusing and releasing internal contents, all while avoiding the immunogenic response [153]. Thus, there is a growing interest in the use of liposomes as drug carriers.

As mentioned, liposomes may be developed to reach variable sizes and internal complexity. The sphere size alters the liposome’s pharmacokinetics and loading capabilities. For instance, smaller molecules may circulate for a longer time, while large spheres may carry a higher therapeutic load [150]. Further variations include unilamellar or multilamellar vesicles, according to the number of internal layers [144].

Liposomes may carry an active pharmaceutical ingredient by passive or active targeting. In passive targeting, the liposome relies solely on the EPR effect to infiltrate the tumor. In this case, the EPR effect is crucial for the selectivity and retention of the loaded active pharmaceutical ingredient. With active targeting, the liposome is enhanced with a tumor-specific ligand, intended to increase specific receptor interaction and endocytosis. However, EPR and receptor density are highly unpredictable and heterogeneous within tumors. Liposomes may also be designed with a stimulus-depended property, such as under magnetic fields, acoustic power, pH, or temperature [152].

Ultrasound-sensitive liposomes, by definition, enhance drug delivery by their susceptibility to mechanical effects under the low-intensity US or thermal effects under the high-intensity US. These liposomes, also known as echogenic liposomes, typically have a gas core. Echogenic liposomes may be prepared by lyophilization with mannitol or by freezing at a high-pressure of gas [154]. An alternative to liposomes with the gas core is to add emulsion droplets that vaporize at the human body’s temperature, termed emulsion liposomes. Similar to the gas core, emulsion liposomes are triggered by ultrasound waves [155]. During the rarefaction phase, the internal pressure drops below the vaporization threshold, and boiling induces collapse. Researchers have demonstrated that triggered delivery of emulsion liposomes is better at lower frequencies [156].

#### 6.2.1. Liposomes for Breast Cancer Treatment

The main challenge in the tumor response to liposomes is the heterogeneous tumor microenvironment. This may include extracellular matrix proteins, matrix metalloproteinase, mesenchymal stromal cells, cancer-associated fibroblasts, and immune cells [157]. To overcome these limitations, a tumor-homing peptide capable of targeting tumor endothelial and stromal cells was explored by Zhu et al. in an MDA-MB-231 tumor spheroid. The authors utilized drug-loaded low-intensity US phase-change liposomes functionalized with a tumor-penetrating peptide which successfully increased tumor penetration, tumor response, and minimized systemic side effects [102].

Extensive preclinical research explored different ligands and formulations to target breast cancer. Awad et al. explored the effects of low-frequency ultrasound in the delivery of a liposome carrying a drug model and tagged with human serum albumin compared to non-targeted liposomes in two different breast cancer cell lines (MDA-MB-231 and MCF-7). This proof-of-concept study demonstrated significantly higher release and tumor cell uptake by targeted liposomes with further increased delivery after ultrasound exposure. The authors also highlighted no significant rise in temperature, which again corroborates the hypothesis that enhanced release is mediated by pore formation led by mechanical effects [103].

Immunoliposomes consist of liposomes coated with tumor-specific antibodies. Elamir et al. compared ultrasound-triggered anti-HER2-antibody-coated liposomes carrying doxorubicin compared to calcein in the treatment of HER2-positive breast tumors. These liposomes were PEGylated to prolong circulation time. The authors demonstrated significant improvement in the drug delivery of immuno-liposomes compared to the non-target and non-sonicated liposomes [104].

A multifunctional approach was chosen instead by Bhardwai et al. They developed a stealth drug delivery system consisting of a nanobubble for ultrasound imaging complexed to a biocompatible thermos- and pH-sensitive liposome (DPPC, DPPE-PEG 2000, and DOPE phospholipids) containing paclitaxel and curcumin. Sulphur hexafluoride gas-filled nanobubbles were prepared from DPPC and TPGS (D-α-tocopherol polyethylene glycol 1000 succinate) to augment cavitation and the EPR effect and from stearic acid to conjugate the NBs to the therapeutic liposome. DPPC makes the liposomes thermosensitive at 41 °C, a temperature that is reached by application of ultrasound, and DOPE instead is sensitive to acidic pH values found at the tumor site. Hyperthermia and low pH allow for the enhancement of drug release by destabilization of the liposome bilayer. The authors showed in an orthotopic TNBC xenograft model of human MDA-MB-231 cells in NOD-SCID mice that enhancing the release of nanoparticles in the tumor site increased the anti-tumor synergist effect and the radiosensitization observed when combining paclitaxel and curcumin [105].

Cressey et al. co-delivered SN-38 (irinotecan’s super-active metabolite) and carboplatin using thermosensitive liposomes (iTSL). Gadolinium lipid conjugate was incorporated into the lipid bilayer to make the iTSL MRI visible. The authors were able to target the delivery of SN-38 and carboplatin to the tumors in TNBC cancer xenografts using focused ultrasound and achieved dramatic inhibition of tumor growth and longer survival of the mice [106]. Using a similar system, M. Amrahl et al. targeted the delivery of doxorubicin in the tumors of mice engrafted with human TNBC cancer using iTSL-encapsulated doxorubicin and focused ultrasound [107].

#### 6.2.2. Strength, Weaknesses, and Open Issues with Liposomes

Liposomes represent a promising candidate in the realm of ultrasound-modulated drug delivery. The versatility of their composition lends them a wide breadth of functionality and physical characteristics [150], which can be adapted to several different applications. The potential for their responsiveness to internal stimuli [158], surface features expanding function and biodistribution profiles [151,152], and their intrinsic ability to appear like endogenous vesicles for the purposes of membrane fusion and reduced immunogenicity make liposomes a powerful therapeutic option [153]. The future direction concerning the development and application of liposomes in theranostics must expand their ability to specifically seek and impact target cell/tissue types by exploring more options for surface functionalization. Additionally, continuing to optimize their circulation times and stability after injection will improve their utility.

### 6.3. Micelles

Micelles are colloidal dispersions consisting of amphiphilic molecules with hydrophilic tails pointing towards the surface, forming a water shell. The hydrophobic head is oriented towards the center. Micelles may carry molecules either in their hydrophobic core or attached to their hydrophilic surface. Compared to liposomes, micelles are smaller in size, but big enough to escape renal excretion and this increases the circulation time [159]. Polymer-based micelles are the most commonly reported type of micelle for ultrasound-triggered drug delivery [160]. These structures are formed by monoblocks, dual-blocks, or tri-blocks of hydrophilic portions composed of hydrophilic poly(ethylene oxide) (PEO) and a hydrophobic core composed of hydrophobic poly(propylene oxide) (PPO) [161]. PEO works similarly to PEG, also commonly used as a hydrophilic block, with its neutral charge aimed at minimizing non-specific interaction to allow for an increased circulation time [162]. Additionally, a discrete pattern of increasing tumor penetrance with a decreasing size of polymeric micelles has been illustrated, with maximal penetrance into even poorly permeable tumor sites being achieved, with the smallest composition of a polymeric micelle (30 nm) [163] representing the general trend of smaller carriers being more capable of utilizing endothelial gaps to extravasate, a distinct advantage over larger, supramicron-sized vehicles such as microbubbles. While the dominant morphology of micellar systems remains spherical, numerous depictions of exotic micellar shapes with varying polymeric shells, such as worm-like/filamentous and rod-like shapes, have been depicted, with some distinct advantages over spherical formulations. For example, filamentous micelles have been shown to have circulation times an order of magnitude longer than their spherical analogs in rodents, with the tradeoff of poorer uptake with longer filaments versus shorter filaments [164]. Recently, rod-shaped PHF-g-(PCL-PEG) polymeric micelles loaded with doxorubicin were shown to have enhanced drug delivery and cellular uptake compared to their spherical counterparts [165]. Additionally, a discrete pattern of increasing tumor penetrance with a decreasing size of polymeric micelles has been illustrated, with maximal penetrance into even poorly permeable tumor sites being achieved with the smallest composition of polymeric micelle (30 nm) [163], representing the general trend of smaller carriers being more capable of utilizing endothelial gaps to extravasate, a distinct advantage over larger, supramicron-sized vehicles such as microbubbles. Several modifications have been employed to improve micellar stability in the bloodstream including copolymerization of an interpenetrating network of thermally responsive acrylates in the hydrophobic micellar core. With this strategy, the micelle’s interpenetrating core expands at room temperature, allowing the introduction of therapeutics into the hydrophobic core. Another modification is to use US for the controlled release of micellar content, which has been studied in preclinical applications with doxorubicin [166].

Studies have demonstrated that the efficiency of US-triggered micellar drug release is inversely related to US frequency and directly related to power density. Longer pulses with short intervals generate faster encapsulation, maintaining an optimal drug concentration between pulses [92]. The processes involved in the US-triggered release of drug-loaded micelles have been shown to involve micellar destruction, cavitating nuclei destruction, micelles reassembly, and drug encapsulation [92].

#### 6.3.1. Micelles for Breast Cancer Treatment

One of the earlier studies using US-triggered chemotherapy-loaded micelles in breast cancer conducted by Howard et al. evaluated a system with paclitaxel encapsulated in polymeric micelles. The authors demonstrated that encapsulated paclitaxel cellular uptake is lower without US compared to the standard clinical formulation. This effect is desired to avoid healthy tissue toxicity, a major concern with paclitaxel. When US was applied, encapsulated paclitaxel produced a 20-fold increase in tumor uptake and inhibited cellular proliferation by nearly 90% [108].

In a more recent study, Chen et al. synthetized nanomicelle drug carriers formulated with PLGA-PEG, loaded with doxorubicin, and tagged with anti-EGFR, which is overexpressed in triple-negative breast cancer. The authors tested solid tumor uptake combined with US-mediated cavitation. For this study, enhanced vascular permeability was induced by using SonoVue^TM^ microbubbles and US in addition to the micelle therapeutic administration. This combined approach aimed to maximize the intra-tumoral uptake and demonstrated better tumor growth suppression at lower drug concentrations [109].

Han et al. designed a new sonosensitizer, PEG-IR780@Ce6, for sonodynamic therapy that is biocompatible and bio-safe. They showed in vitro and in vivo in TNBC cells an improved uptake of PEG-IR780@Ce6 under US irradiation and the generation of higher levels of reactive oxygen species when compared to IR780 and free Ce6 alone or combined with US. This led to an increase in anti-cancer effects. Additionally, PEG-IR780@Ce6 inhibited TNBC cell migration and invasion and suppressed the expression of MMP-2 and MMP-9, potentially suppressing metastasis [110].

#### 6.3.2. Strength, Weaknesses, and Open Issues with Liposomes

Micelles have shown some promise in their applications as described above, and they possess some strengths over other vehicles. Namely, they have a smaller size but remain large enough to avoid renal excretion to improve biodistribution and increase circulation time [159]. Nanocarriers have some challenges. The critical micellar concentration (CMC) is the concentration threshold for micelles to form [167]. This comprises one of the main challenges in the use of micelles because of their instability when diluted into the bloodstream, potentially releasing the therapeutic prematurely. For instance, the concentration of micellar needed to maintain the micellar concentration above its CMC would not be tolerable in humans. Another challenge, shared with all nanocarriers, is recognition by the immune system. Micelles made of polymers are the most commonly used and have the advantage of a lower CMC compared to surfactant micelles [168]. These are also usually coated with PEO, as mentioned earlier, which prevents recognition by the immune system. The generation of micellar systems that improve stability and further improve their avoidance of immunorecognition is needed.

### 6.4. Polymeric Nanoparticles

Polymeric nanoparticles (NPs) represent an ideal drug delivery system because they are biomimetic, biocompatible, biodegradable, and water-soluble. Natural (e.g., alginate, chitosan, gelatin, and albumin) and synthetic (e.g., poly (lactic acid) (PLA), poly(e-caprolactone) (PLC) and poly (lactic-co-glycolic acid) (PLGA),) polymers can be used to produce nanoparticles. The synthesis of the different types of polymeric NPs can be achieved by microfluidics [169], nanoprecipitation [170], emulsification [171], and ionic gelation [172]. Generally, polymeric nanoparticles inhabit a size range between 100 and 300 nanometers, but lower-sized formulations have been generated. These carriers come in two general morphologies: the nanosphere and the nanocapsule. Nanospheres are constituted by a solid structure composed of the constituent polymer that constitutes the shell and the matrix, whereas nanocapsules have a very thin polymeric envelope that covers a liquid phase, frequently an oily core [173]. This lends a robust versatility to this class of vehicle, as small molecules of varying hydrophilicity can be associated with either the constituent polymer core or with the shell itself in the case of capsules. Capsules also have an advantage as the hydrophobic oil core is readily available to incorporate hydrophobic small molecules. Polymer variation can also be leveraged to optimize the delivery of charged macromolecules, achieved with either cationic polymer moieties complexing with anionic molecules such as RNA or direct conjugation of polymer units with the macromolecules themselves or through a cationic intermediate moiety [174].

The drugs can be bound to the surface or conjugated to the polymer, encapsulated in the hydrophobic core like in the case of nanocapsules, or embedded in the matrix like for the nanospheres [169,175,176,177,178,179]. Because of the sheer versatility of their therapeutic-binding ability owing to their readily customizable composition, numerous classes of therapeutics, from hydrophobic small molecules, which historically have been notoriously difficult to distribute, to charged macromolecules, have the potential to be utilized. Furthermore, they can be formulated to be able to precisely control the loading and the kinetics of release of therapeutics [180,181]. NPs can be engineered to present PEG on the surface, PEGlyation, with the goal to avoid recognition by phagocytic mononuclear cells. However, this is not completely achieved because it has been reported that exposure to PEG can lead to the production of antibodies anti-PEG and clearance of PEGylated NPs [182,183].

#### 6.4.1. Polymeric Nanoparticles in Breast Cancer

Suicide gene therapy, or gene-directed enzyme prodrug therapy (GDEPT), is a common approach to treating solid tumors. Devulapally et al. by exploiting this platform synthesized a biodegradable PLGA/PEI nanoparticles (polyethylene glycol (PEG)ylated-poly (lactic-co-glycolic acid)/polyethyleneimine) complexed to plasmid vectors expressing the HSV1-sr39TK-NTR (TK–NTR) fusion gene under a tumor-specific survivin promoter. Clinically translatable US-MBs (Bracco)-mediated drug delivery was used to enhance the delivery of NPs and plasmid to the tumor site in TNBC xenografts in vivo. The authors observed a further reduction in tumor growth when US-MB treatment was added to the combination of PLGA/PEI NPs with the TK–NTR fusion gene and prodrugs (GCV/CB1954) [111].

Carlisle et al. complexed adenoviruses with a N-(2-hydroxypropyl)methacrylamide polymer to obtain a stealth system to protect from liver sequestration and toxicity and to increase blood half-life. They injected systemically the polymer-coated oncolytic virus together with SonoVue microbubbles in mice bearing xenografts of the human breast cancer cell line ZR-75-1. They treated tumors with focused ultrasound 0.5 MHz at peak rarefactional pressure of 1.2 MPa and showed an increase of 30-fold in viral infection and reduction of tumor growth [112].

Kim et al. synthesized a pH-sensitive, reduced albumin nanoparticle loaded with doxorubicin to use in combination with focused ultrasound treatment. The ultrasound application allowed for a targeted accumulation of the nanoparticles in the tumor site, and the acidic pH in the tumor microenvironment and inside the cells for example in lysosomes led to a complete release of doxorubicin and increased therapeutic effect in the TNBC xenograft in mice [113].

#### 6.4.2. Strength, Weaknesses, and Open Issues with Polymeric Nanoparticles

A significant advantage illustrated over the development of polymeric NPs is seen with how highly customizable they are. For instance, polymeric NPs have been developed with surface functionalization utilizing a variety of moieties, including site-targeting as well as metallic moieties that have a direct cytotoxic, genotoxic, and photoacoustic capacity [180]. This represents an exciting potential for expanding the physical and biochemical profiles of these agents, which increases the scope of their applications with subsequent research. Major limitations of these systems, particularly those with metallic surface moieties, lie in the potential for off-target toxicities. Additionally, as is the case with any vehicle that uses polymeric materials, the number of well-described polymers available for use as drug delivery systems is somewhat limited, though ongoing work is seeing this repertoire expand rapidly [184]. Characterization of each specific system’s toxicity and biodistribution will be required to ensure safety in the translational space and beyond.

### 6.5. Nanoemulsion/Droplets

Emulsions are kinetically stable but thermodynamically unstable, biphasic liquid–liquid dispersions of variable sizes (microemulsion and nanoemulsion) consisting of two immiscible liquids, one suspended into another [185]. Nanoemulsions are typically under 200 nm in diameter, with some definitions considering both 500 and 100 nm to be the upper limit. These are most commonly formed by oil and water combinations further stabilized by emulsifiers. The addition of low surfactant concentration is what turns the emulsion thermos-responsive [186]. Emulsifiers are ideally used in the minimum concentration needed to maintain the interfacial tension. Emulsions are derived from the addition of stabilization components to the historically previously described colloids [187].

Kinetic stability allows for stable drug delivery formulations. Under a thermodynamic stimulus, emulsions are prone to destabilization and liquid emulsion, a characteristic explored with US-triggered drug delivery. Reported methods to produce nanoemulsions include high-energy methods such as high-speed homogenization, ultrasonication, high-pressure homogenization, microfluidic and membrane methods, and low-energy methods, such as phase inversion temperature and emulsion point inversion [188]. The literature has demonstrated that high-energy methods tend to be less efficient and most of the applied energy is dissipated into heat, thus low-energy methods are currently preferred [189].

The oil–aqueous mixture and the surfactant concentration must be appropriately selected according to the intended carried molecule, which is usually loaded into the oil core [190]. Cellular nanoemulsion uptake mechanisms include direct paracellular or transcellular transport. Non-US-enhanced nanoemulsion delivery has been described through different administration routes (intranasal, ophthalmic, oral, topical, and transdermal) and some are available for clinical use [191]. US irradiation plays synergistic effects to enhance nanoemulsion-based drug delivery and enhanced imaging. This effect is allowed by the US-triggered liquid-to-gas transition of nanoemulsions once the vaporization threshold is reached [192]. The liquid-to-bubble transition increases the interior volume and ultimately generates vesicle rupture and local drug release. Intrinsic droplet properties influence the acoustic pressure required for transition including the size, type of formulation, pressure, and temperature of the medium. After being vaporized, these nanoemulsions, now microbubbles, improve ultrasound tissue echogenicity by increased backscattering with the same mechanism as commercially available MBs [193]. While emulsions excel at delivering hydrophobic drugs due to their highly lipophilic core, creative rational design of surfactant moieties to encompass hydrophilic/ionic polymers or other functional conjugates allows for a robust expansion of the nanoemulsions’ versatility. Cationic nanoemulsions have already been shown to be able to deliver RNA in the context of early-in-development nonviral vaccines [194], as well as immunomodulatory small molecules to serve as adjuvants for vaccination against melanoma, lung cancer, and cervical cancer in tumor models [195,196]. The scope of nanoemulsions’ therapeutic repertoire will likely continue to expand with the increasing rational design of its surface profile and payload capacity.

Rapoport et al. compared the effects of a micellar formulation or nanodroplet formulation plus ultrasound and noted a significant difference between the ultrasound-treated and untreated groups. Furthermore, increased accumulation of intravenously administered nanodroplets was demonstrated by increased echogenicity in the tumor ultrasound images. The same groups reported in a different study an inverse relationship between the size of droplets and vaporization threshold. This effect is caused by increased Laplace pressure and thus increased boiling point in smaller nanoemulsions. Additionally, the ADV threshold is typically lower than the inertial cavitation, which may be another advantage of nanoemulsions compared to MBs when capsule disruption and drug delivery are desired [197].

In a similar fashion to acoustic vaporization of phase transition vectors described above, optical droplet vaporization (ODV) is receiving attention as an alternative phase-transition technique for imaging and therapeutics, and it has received increasing attention with the development of a growing number of vehicles as an alternative or complement to ultrasound-induced vaporization techniques. This technique leverages the ability of metallic, usually gold or silver, nanoparticles acting as chromophores to absorb optical energy and transform it into a local temperature increase. This precipitates the vaporization of perfluorocarbon or other liquid phases loaded into nanoparticles of a variety of compositions to rapidly expand and generate acoustic forces on local tissues similar to those experienced with conventional ADV, allowing for contrast enhancement or drug delivery [198].

Dove et al. [199] illustrated the feasibility and utility of optically responsive phase transition vehicles in their work on gold-nanoparticle-conjugated nanodroplets. Gold nanoparticles were conjugated to the surface of 1,2-diarachidoyl-sn-glycero-3-phosphocholine (DAPC), 1,2-distearoyl-sn-glycero-3-phosphorethanolamine-N-[methoxy (polyethylene glycol) 2000] (DSPE-PEG2K), and 1,2-distearoyl-sn-glycero-3-phospho-ethanolamine-N [biotinyl (plyethylene glycol)-2000] (DSPE-PEG2K-B) microbubbles with a variety of perfluorocarbon cores, octafluoropropane (C3F8), decafluorobutane (C4F10), and dodecafluoropentane (C5F12) to generate microbubbles that undergo vaporization when irradiated with a laser. The microbubbles containing a C3F8 core had an optical vaporization threshold 9-fold lower than C5F12, which has been more commonly used when approaching photoacoustic vaporization. This development represents progress in lowering the required energy density to functionalize phase-change vectors that are responsive to optical stimuli, which further increases its potential for applications in vivo. Further proof of concept and its potential utility was highlighted by Toumia et al. [200]. Diacetylene polymeric shells 10,12-pentacosadiynoic acid (PCDA) encapsulating perfluoropentane and perfluorobutane nanodroplets represent a hybrid between lipidic and polymeric shelled nanodroplets that undergo phase transition via ADV. These moieties were readily functionalized with RGD, described earlier, as well as a fluorophore and were shown to possess good biocompatibility, modification potential, and adhesion to target fibroblast in vitro [200].

Lajoinie et al. [201] designed two polymeric capsules encapsulating various volatile oils that underwent optically inducible vaporization, one made from polymethylmethacrylate (PMMA) loaded with hexadecane and the other formed from poly (lactic-co-glycolic acid) (Resomer) microparticle containing perfluropentane (PFP) oil. The transition from microparticle to microbubble after laser-induced vaporization induced poration as well as human endothelial cell death in vitro. The Resomer-PFP microparticle has a lower activation threshold, and thus requires a lower laser intensity than PMMA with hexadecane to induce vaporization. In both microparticles, vaporization induced poration and subsequent cell death, with larger resulting bubbles resulting in 100% poration probability with larger bubbles. This technique is limited largely by a low penetration depth, a few centimeters, and this presents a major barrier to using this technique for enhancing drug delivery as well as inducing localized cell death.

#### 6.5.1. Nanoemulsions/Nanodroplets for Breast Cancer Treatment

Nanoemulsions have been used to deliver lipophilic drugs. Prasad et al. reported on the anti-tumor efficacy of lecithin-based curcumin encapsulated nanoemulsions and exposed to ultrasound with or without microbubbles. The system was tested in triple-negative breast cancer cell lines (MDA-MB-231) and melanoma cells (B16F10) [114]. The use of ultrasound combined with microbubbles significantly increased tumor cytotoxicity in vitro by 100- and 64-fold in breast and melanoma cells. The same trend was then demonstrated on melanoma subcutaneous tumor xenografts in mice with enhanced tumor growth inhibition in the tumors undergoing ultrasound.

Baghbani et al. synthesized multifunctional nanodroplets which were ultrasound responsive with the addition of alginate-stabilized perfluorohexane loaded with doxorubicin. These multifunctional nanodroplets when further enhanced by sonication demonstrated significant tumor regression resulting from on-demand drug delivery. This was demonstrated by a 5.2-fold higher doxorubicin concentration in tumor tissue that underwent ultrasound compared to non-sonicated tissue. These theranostic particles also showed successful increased echogenicity under ultrasound imaging. Additionally, the authors demonstrated a marked decrease rate of cardiotoxicity in the US-enhanced nanodroplet-encapsulated doxorubicin group compared to the non-encapsulated drug [115].

Rapoport et al. reported the properties of perfluoro-15-crown-5-ether (PFCE) loaded with paclitaxel which displays ultrasound and fluorine MR contrast properties. Ultrasound triggered a reversible droplet-to-bubble transition with the microbubbles formed by acoustic vaporization undergoing stable cavitation. The use of PFCE nanoemulsions loaded with paclitaxel and in combination with US allowed to achieve notable therapeutic effects such as complete tumor regression and metastasis suppression in pancreatic and breast cancer [116].

Multifunctional perfluorohexane nanoemulsions coupled to silica-coated gold nanoparticles (PFH-NEs-scAuNPs) have demonstrated efficient chemotherapeutic loading capability as shown with doxorubicin, 5-fluorouracil, and paclitaxel. This formulation has shown utility in photoacoustic, ultrasound, and fluorescence imaging in vitro and in vivo. Moreover, the local nanoemulsion’s expansion and rupture can be used for tumor treatment, as shown by Fernandes et al. in 4T1 tumor-bearing mice [117].

Wang et al. [202] further expanded upon the utility of ODV in phase-transition vectors in their experiment by utilizing silica-coated gold nanorods (GNR) and perfluorohexane (C6F14) into PLGA-PEG nanoparticles, which were further functionalized by surface conjugation of Herceptin antibodies. These particles were evaluated for site-specific accumulation and therapeutic efficacy against MDA-MB-231 (HER2- negative) and BT474 (HER2-positive) xenograft mouse models. These nanoparticles had 19x greater binding efficacy to HER2-positive cell lines than to the HER2-negative cell lines, and histological analysis revealed tissue damage when the GNR-PLGA-PEG nanoparticles were exposed to lasers versus a control or unfunctionalized nanoparticles. This indicates the potential for surface functionalized, optically vaporizable nanoparticles use in the treatment of HER2-positive breast cancers or malignancies with surface features amenable to predictable antibody targeting.

#### 6.5.2. Strength, Weaknesses, and Open Issues with Nanoemulsions/Nanodroplets

When compared to microbubbles, nanoemulsions’ pre-transition small sizes allow for better tissue penetrance via EPR, increasing the delivery of any intended payloads as well as increasing tissue echogenicity on ultrasound. This effect is allowed by the US-triggered liquid-to-gas transition of nanoemulsions once the vaporization threshold is reached [192]. The potentially lower energy of ADV, when compared to MBs, means easier capsule disruption and drug delivery since energy transmission or off-target effects of higher energy irradiation can be reduced. Additionally, the drug payload of nanoemulsions is limited by the ultrasound response [203], which fundamentally limits the quantity of payload able to be delivered. Finally, the manufacture of many of these vehicles is very costly and requires specialized equipment [204]. This presents a problem when considering production scale or widespread adoption, and this will necessitate the development of manufacturing conditions that are more readily utilizable. Several future directions of research exist characterizing the stability, biodistribution, and toxicity of emerging systems, especially those with metallic components with known local and systemic toxicities, and are paramount in being able to advance and optimize these technologies into future in vivo, translational, or eventually preclinical spaces.

## 7. Biocompatibility

The compositions of ultrasound-responsive vehicles are highly diverse, and so too are their biodistribution and toxicity profiles. With increasing surface moieties used in the functionalization of many types of vehicles, immunorecognition is a significant hurdle to maximizing bioavailability. One of the most widely used methods of escaping immune detection as well as improving circulation dynamics is PEGylation [205]. Surface modification with PEGylation has also been shown to reduce deposition into the reticular endothelium system of the liver, lungs, and spleen [206]. Another method used to reduce toxicity and improve target site delivery is using high-affinity ligands. Conjugation of high-affinity ligands, such as RGD which preferentially binds to upregulated integrins frequently seen in neovascular processes, increases the concentration of actively delivered therapies from nanoparticles and reduces systemic toxicities, likely from reducing accumulation within non-targeted tissues [207]. This reduced toxicity was also confirmed in RGD-conjugated microbubbles in a murine model where adequate post-treatment growth, no mortality, and only mild reversible biological effects were observed [208]. This highlights the strength of using active targeting with specific surface functionalization to reduce systemic toxicity.

Not only does the attachment of surface moieties for functional purposes play a role in their biocompatibility, but so too does the actual composition of the carrier shell. Protein-coated carriers and many polymeric carriers have been used extensively because of their tendency for excellent biocompatibility and biodegradability. Alginate, chitosan, gelatin, and albumin are among the most common proteins to encapsulate polymeric nanoparticles, and they have favorable biocompatibility [209]. Polymeric micelles constituted from methoxy-poly (ethylene glycol)-poly(d,l-lactide) (MPEG-PLA) were found to be nontoxic to the human reticular endothelial system [210]. In an analogous manner, a stealth system has been generated by coating oncolytic adenovirus with (2-hydroxypropyl)methacrylamide [112]. However, there is evidence that some cationic polymeric shells in liposomes and micelles have in vivo toxicity to lung and liver tissue [211,212]. This is due to the enhanced production of reactive oxygen species as well as more extensive cell surface–carrier interaction resulting in enhanced uptake. In contrast to cationic polymeric micelles, polymeric microbubbles constructed with PVA have been shown to undergo adequate elimination without discernable defects both in vivo and in vitro [213]. Much in the same way, several studies have shown that nanobubbles have limited in vivo toxicity, owing to the fact that the perfluorocarbon payload and phospholipid shells frequently employed are generally non-toxic [214,215]. Biocompatibility is not mediated only by the chemical composition or surface features of a given carrier. The physical characteristics of the carrier itself also greatly impact the toxicity profile of the system. For instance, PLGA-PEG nanoparticles have been shown to have widely different cytotoxicity dependent on the shell’s physical shape. Zhang et al. illustrated that needle-shaped PLGA-PEG particles induced apoptosis significantly more than their spherical counterparts of the same chemical composition, which is thought to occur because of differential disruption of lysosomal membranes [216]. Size has been illustrated to impact microbubble uptake and target accumulation and is much of the basis for EPR, with smaller sizes allowing for target tissue accumulation [120]. Size also plays a role in determining systemic toxicities. For instance, PDLA-PEG nanoemulsions and nanomicelles loaded with paclitaxel were shown to have different hematological toxicities in mice, with nanoemulsions of 200 nm to 1000 nm exhibiting significantly less hematological toxicity than their nanomicelle counterpart, with dimensions of only 20 to 100 nm. Gold-based nanoparticles also demonstrate enhanced cellular uptake at a smaller size [217,218] although conflicting toxicity determinations regarding size trends exist within the literature. Ultimately, the biocompatibility of the systems discussed above and those being developed are exquisitely complex and rely on a myriad of interactions between shell composition and surface features–cell interaction, size, shape, and stability. Future work towards establishing effective, safe theranostic systems will require careful balancing of the physiochemical properties of the given carrier to optimize drug delivery and stimuli responsiveness while minimizing off-target toxicities, and this will be aided by elucidating the toxicities and pharmacokinetics of emerging carrier–agent combinations.

## 8. Comparison of Lipidic and Polymeric Delivery Vehicles to Other Promising Nanoparticle Systems

Nanoparticles constructed of metal oxides are part of a class of drug delivery vehicles that are quite diverse in morphology, formulations, and applications. Metal oxide nanoparticles can be formulated to produce sheets, nanotubes, multifaceted nanoparticles, and even nanoflowers, all of which have very diverse distribution and internalization characteristics [219]. Additionally, metal oxide nanoparticles reveal new opportunities for the induction of drug delivery and imaging modalities apart from ultrasound. Magnetic-directed heating of iron oxide nanoparticles to temperatures that can ablate tumor tissue is a prime example of an alternative therapeutic and induction strategy not seen with purely polymeric or amphiphilic vehicles without significant surface modulation [220]. However, many of the metal oxide nanoparticles are intrinsically toxic, owing to their generation of ROS and damage to the genome and cytoplasmic structures; the size, morphology, and metal composition are highly variable and dictate the extent of toxicity when exposed to non-target tissue [221]. In contrast, many of the constituent polymers and lipids characterized above are generally well tolerated and serve as a close approximation to native tissue, with exceptions depending on size and surface features notwithstanding. There is undeniable potential for unintended toxicities, but the wealth of understanding of biocompatible polymers and their derivatives makes them generally easier to approach in anticipating untoward effects. In spite of this, many next-generation vehicles can utilize metal oxide nanoparticle conjugates and complexes to enhance their therapeutic utility by expanding therapeutic profiles or even opening new avenues for temporally and spatially selective drug delivery, as is the case of paramagnetic iron oxide and other metal oxide functionalized vehicles generating phase-change under magnetic stimulus [222,223]. There is much benefit in utilizing the classes of delivery vehicles synergistically.

Mesoporous silica nanoparticles (MSN)s represent another promising candidate in site-directed drug delivery. They possess a high degree of customizability in morphology and boast an impressive surface area that allows for an increased interface with the tissue environment, in addition to pores that can be customized for the highly controlled release of therapeutics [224]. In a similar fashion to the polymeric and amphiphilic classes denoted above, rational design of surface features, such as hydrophobicity and porosity, can be used to optimize their behaviors in vivo by impacting distribution and biocompatibility [225]. MSNs also have the capacity to be inducible in their drug delivery, both reversibly and irreversibly modulating their pores’ communication with the interstitium, with external stimuli such as ultrasound [226]. Many of these features are quite reminiscent of the highly customizable surfaces and ultrasound inducibility of polymeric carriers, with both MSNs and the numerous delivery vehicles discussed above being able to undergo customization to optimize their biological behaviors and drug delivery in vivo. The same concerns of off-target toxicities and possible unforeseen bioaccumulation that come with all nanoparticles with potentially wide distribution also remain. Ultimately, while different in that MSNs are not organically derived as is the case of polymeric or lipidic carriers, they serve as a reasonable and promising avenue.

Extracellular vesicles are a highly heterogenous class of carrier characterized by a bilayer membrane, quite similar in their general morphology to liposomes [227]. In spite of their similarities to liposomes, extracellular vesicles have a much higher degree of surface complexity owing to their rich lipid bilayer components, which ultimately depends on their manufacturing technique. They possess the advantage of generally being very similar to endogenous vesicles and much more native-appearing than conventional liposomes, which can greatly increase confidence in their biocompatibility as well as limit nonspecific interaction that is seen with synthetic or semisynthetic liposomes. However, when compared to engineered liposomes, extracellular vesicles present problems of surface composition heterogeneity, which is a challenge to predicting consistent biocompatibility, bioavailability, and on-target delivery [228]. Exploring the overlap between the dichotomy of liposomes and EVs by adapting the surface richness of EVs granting the ability to access many endogenous pathways for enhanced distribution and uptake in vivo with the measure of composition control and predictability of liposomes represents an exciting prospective for the improvement of bilayer-derived delivery vehicles.

## 9. Conclusions

Ultrasound-enhanced delivery of cancer therapeutics from micro-nanobubbles and other acoustic-sensitive carriers has emerged as a feasible method for increasing the accumulation of drugs/genes to the targeted tumor region mainly by improving the EPR effect. The possibility of synthesizing these nanoparticles with biomaterials makes the system potentially highly safe for human applications.

One of the limitations of this system is the reduced drug/genes encapsulation capabilities of US-responsive materials especially highly echogenic micro-nanobubbles with a gas core. Other nanomaterials that have higher loading efficiency are less responsive and higher US frequencies are necessary to release the therapeutics which can cause damage to the healthy surrounding tissues [120]. A way to overcome this obstacle, in the case of gene therapy, would be to develop ultrasound-responsive systems that can either encapsulate or shield and deliver viral vectors which display high and sustained expression of the therapeutic gene, such as adeno-associated viruses [229] or to use oncolytic viruses that can conditionally replicate in the tumor and express a transgene [139].

As previously discussed, different levels of thermogenesis can be reached by ultrasound depending on the use of low, high-frequency, or focused ultrasound. Even if the thermal effect can be exploited to design carriers that are for instance temperature sensitive, US can induce tissue damages that are proportional to the intensity and duration of the irradiation. At the same time, targeted permanent tissue damage has been exploited to therapeutically intervene on the tumor mass and induce ablation by applying focused ultrasound. Additionally, focused ultrasound has also been employed to transiently open the blood–brain barrier (BBB) to improve the delivery of drugs to the brain. A phase I clinical trial was conducted by the Sunnybrook team which showed that transient opening of the BBB with focused ultrasound enhanced the delivery of trastuzumab to breast cancer metastasis in the brains of HER-2 positive patients [230].

Despite the amount of preclinical data showing the possibility of effectively guiding and increasing drug and gene transfer in tumors, not enough clinical trials have been initiated especially in the therapeutic area of breast cancer and more importantly triple-negative breast cancer which nowadays still have limited therapeutic options. Taking into consideration that ultrasound represents in the clinical setting one of the main imaging tools for the screening, diagnosis, staging, surgical planning, and surveillance of breast cancer, it would be extremely important and advantageous to develop its therapeutic capabilities and maximize its clinical application in combination with ultrasound-sensitive carriers to deliver therapeutics to the tumor and avoid non-specific targeting and systemic toxic effects.

## Figures and Tables

**Table 1:** Preclinical studies using US responsive carriers for US-enhanced delivery of drugs/genes for the treatment of breast cancer.

Nanoparticle	**Ligand**	**Therapeutic**	**Effect**	**Refs.**
Micro/Nanobubble				
Microbubbles stabilized by PEGylated nanoparticles of poly (butyl cyanoacrylate) PBCA polymer	-	Cabazitaxel	A rate of 2.3 tumor uptake improvement after bubbles destruction by focused ultrasound	[98]
Microbubble (RGD-PEG-DPPE, DPPC containing SF6 gas)	RGD	Paclitaxel	US increased drug accumulation in TNBC-targeted tumors in vitro	[99]
Nanobubbles (DPCC, DSPE-PEG2000-MAL, DPPA, PEG-40 stereate)	CPP	siEGFR	Reduction in EGFR mRNA and protein levels, reduction in cell proliferation in vitro, and inhibition of tumor xenografts growth in vivo	[99]
Perfluoropropane filled nanobubbles (DSPC and DSPE-PEG2000)	CPP	LINC00511-siRNA cisplatin	Reduction in LINC00511 expression and increased sensitivity to cisplatin	[100]
Microbubble/microdroplet clusters (PS101)	-	Doxil	Increased drug accumulation in the tumor in orthotopic human TNBC xenografts	[101]
**Liposome**				
US phase-change liposomes	Tumor homing peptide	-	Targeting tumor endothelial and stromal cells	[102]
Liposome	Human serum albumin	Model drug (calcein)	Increased albumin uptake in tumor cells compared to the healthy cells	[103]
PEGylated immunoliposomes	Anti-HER2	Doxorubicin	Increase in doxorubicin uptake	[104]
Thermo and pH sensitive liposome (DPPC, DPPE-PEG 2000, and DOPE phospholipids)	-	Paclitaxel and Curcumin	Increased anti-tumor synergist effect and radiosensitization of paclitaxel and curcumin in vivo	[105]
Thermosensitive liposomes (iTSL)	-	SN-38 carboplatin	Targeted delivery of SN-38 and carboplatin, inhibition of tumor growth, and 2.5 × longer survival times	[106]
Thermosensitive liposomes (iTSL)	-	Doxorubicin	Targeted delivery of doxorubicin, inhibition of tumors	[107]
**Micelle**				
Polymeric micelle	-	Paclitaxel	Twenty-fold increase in tumor uptake and inhibition of cellular proliferation by nearly 90%	[108]
Nanomicelles (PLGA-PEG) SonoVue	Anti-EGFR	Doxorubicin	Maximized intra-tumoral uptake and demonstrated better tumor growth suppression at lower drug concentrations	[109]
PEG-IR780@Ce6	-	IR780 Ce6	Increase of ROS in vitro and in vivo, inhibition of migration and invasion, and inhibition of tumor cells growth in vivo	[110]
**Nanoparticle**				
Nanoparticle (PLGA/PEI) Bracco MBs	None	Plasmid TK–NTR fusion and gene survivin promoter GCV/CB1954	MBs-US enhanced the delivery of NPs TK–NTR and increased the therapeutic effect in vivo	[111]
Adenoviruses- N-(2-hydroxypropyl)methacrylamide polymer SonoVue	-	Adenoviruses	Twenty-fold decrease in viral infection and reduction of tumor growth	[112]
Reduced albumin	-	Doxorubicin	Increase in NPs accumulation and therapeutic effect in vivo	[113]
**Nanodroplet/Nanoemulsion**				
Lecithin-based nanoemulsion microbubbles	-	Curcumin	US increased cytotoxicity of curcumin in breast cancer in vitro in and melanoma in vitro and in vivo	[114]
Alginate-stabilized perfluorohexane multifunctional droplets	-	Doxorubicin	A 5.2-fold higher doxorubicin concentration and decreased cardiotoxicity in tumor tissue that underwent US treatment	[115]
Perfluoro-15-crown-5-ether (PFCE)	-	Paclitaxel	Tumor regression in vivo	[116]
Perfluorohexane nanoemulsions coupled to silica-coated gold nanoparticles		Doxorubicin, 5-fluorouracel Paclitaxel	Multi-modality bio-imaging and local therapy	[117]
